# Determinants of formal care use and expenses among in-home elderly in Jing’an district, Shanghai, China

**DOI:** 10.1371/journal.pone.0176548

**Published:** 2017-04-27

**Authors:** Fen Li, Xinye Fang, Jiechun Gao, Hansheng Ding, Changying Wang, Chunyan Xie, Yitong Yang, Chunlin Jin

**Affiliations:** 1 School of Public Health, Fudan University, Shanghai, P.R.China; 2 Shanghai Health Development Research Center, Shanghai, P.R.China; 3 Shanghai Medical Information Center, Shanghai, P.R.China; 4 Shanghai University of Finance and Economics, Shanghai, P.R.China; 5 Shanghai Population Development Research Center, Shanghai, P.R.China; Universidade Federal do Rio de Janeiro, BRAZIL

## Abstract

The need for formal care among the elderly population has been increasing due to their greater longevity and the evolution of family structure. We examined the determinants of the use and expenses of formal care among in-home elderly adults in Shanghai. A two-part model based on the data from the Shanghai Long-Term Care Needs Assessment Questionnaire was applied. A total of 8428 participants responded in 2014 and 7100 were followed up in 2015. The determinants of the probability of using formal care were analyzed in the first part of the model and the determinants of formal care expenses were analyzed in the second part. Demographic indicators, living arrangements, physical health status, and care type in 2014 were selected as independent variables. We found that individuals of older age; women; those with higher Activities of Daily Living (ADL) scores; those without spouse; those with higher income; those suffering from stroke, dementia, lower limb fracture, or advanced tumor; and those with previous experience of formal and informal care were more likely to receive formal care in 2015. Furthermore, age, income and formal care fee in 2014 were significant predictors of formal care expenses in 2015. Taken together, the results showed that formal care provision in Shanghai was not determined by ADL scores, but was instead more related to income. This implied an inappropriate distribution of formal care among elderly population in Shanghai. Additionally, it appeared difficult for the elderly to quit the formal care once they begun to use it. These results highlighted the importance of assessing the need for formal care, and suggested that the government offer guidance on formal care use for the elderly.

## Introduction

In 2015, the number of the household residents aged 60 and above reached 4.36 million in Shanghai and the average life expectancy of the total household population was 82.75 years. Approximately 2.72% of elderly adults were reported as requiring complete help and need help for most of the tasks in 2014 [1]. Continuous efforts have been made by the government to develop a sustainable long-term care system, among which home-based care provided by formal and informal caregivers has been strongly advocated. Formal care refers to professionally organized paid assistance whereas informal care refers to unpaid assistance provided by informal networks or unlicensed personnel; the majority of long-term care is informal care [2–4]. However, it is believed that the need for formal care might have increased in recent years, in line with the greater longevity and changes in family structure [5].

A number of studies have examined the factors associated with the use and expenses of formal care. These relevant factors can be classified into three categories: (1) demographic and socioeconomic characteristics, including age, gender, education, ethnicity, marital status, number of children, and household income; (2) health conditions, including Activities of Daily Living (ADL) and Instrumental Activities of Daily Living(IADL), self-reported health and chronic conditions; and (3) availability of formal and informal care services, such as living arrangements. Among them, ADL limitations, which are an important index of functional impairment, have been recognized as a significant predictor of formal care reception [6–8]. This is understandable, as a greater degree of functional impairment would by nature imply a greater degree of dependency, thereby leading to greater use of and expenses in formal care. A higher economic status is also associated with a greater probability of using formal care [9, 10]. Other variables such as age, gender, self-reported health, and chronic diseases were found to be significantly related in some studies [5, 6, 11–15]. The substitution or complement effects between formal and informal care have been discussed for a long time [16–21].

However, most of these studies were conducted in western countries, and thus have limited significance to China. In the present study, we sought to identify the factors influencing the use and expenses of formal care among in-home elderly adults in Shanghai. We expected that it would be valuable to policymakers aiming to improve the provision of long-term care.

## Methods

### Sample and questionnaire

This study was embedded within the Shanghai Long-Term Care Needs Assessment Questionnaire (SLTNAQ), a cohort survey conducted in Jing’an district, Shanghai. The proportion of the elderly aged 60 years or above in Jing’an was largest among all districts of Shanghai, at 33.9%, in 2015. The SLTNAQ is a longitudinal survey, designed to examine the long-term care needs of the elderly. The variables assessed by the SLTNAQ include demographic indicators (i.e. age, gender, and income), living arrangements, ADL, IADL, mental health status, cognitive status, physical status and a clinical diagnosis provided by general practitioners. From among five sub-districts in Jing’an, Jiangning Road was randomly selected and sampled. Among the 17000 elderly living in Jiangning Road, 8500 residents aged over 60 were randomly selected and observed in 2014. Of these, 8428 in-home individuals responded to the questionnaire. 7100 of them were followed up in 2015. We found no difference in the descriptive characteristics between respondents observed in 2014 and those followed up in 2015 (*p*>0.05). Only 0.48% of the respondents who lived at home in 2014 had transferred into an institutions by 2015.

### Dependent variables

Dependent variables were the probability of formal care use and formal care expenses. All paid formal care provided at home was considered. Considering the three main kinds of formal care in Shanghai, the expenses included the amount of money paid to professional workers affiliated with public health institutions, nursing workers affiliated with private companies and self-hired housemaids.

### Independent variables

#### Demographic indicators and living arrangements

Demographic and socioeconomic factors were considered as potential determinants of formal care use. These variables included age, gender, monthly income, marital status (with/without spouse), and with/without children. Living arrangements were determined as whether they live alone or not. Age and ADL were included in the model as continuous variables.

#### Physical health status

Physical health status was determined using the objective indicators of ADL (scored from 0 to 20) and whether participants have certain chronic conditions, as well as the subjective indicator of self-reported health. ADL summarizes an individual’s overall performance in feeding, bathing, dressing, continence, going to the toilet, and transferring. A lower ADL score indicates greater functional impairment and a greater level of dependency [22, 23]. A total of 17 chronic diseases were surveyed in the SLTNAQ, based on the most common diseases found among inpatients in nursing homes. In the present study, we only selected the diseases that require a considerable amount of care as independent variables, including hypertension, coronary heart disease, stroke, diabetes, advanced tumor, lower limb fracture, and dementia [24]. For elderly adults with dementia, their family members or main caregivers responded on their behalf. According to participants’ responses to the self-reported health indicator, they were divided into two groups: healthy (i.e. those who reported themselves as having very good, good, or fair health) and unhealthy (i.e., those who reported themselves as having bad or very bad health).

#### Care type used in 2014

The types of care used in 2014 included formal and informal care. Participants were classified into four groups based on their answers: with/without formal care and with/without informal care. The categories were not mutually exclusive; in other words, respondents with formal care could have informal care simultaneously.

### Quality control

Three steps were taken to ensure the quality of the data. First, interviewers double-checked each questionnaire at the end of the interview to determine whether there were any blank spaces. Second, the logical relations and missing data in the questionnaire were determined and reconfirmed with the interviewees via telephone if major problems discovered. A professional company was hired to input the data and establish a database. Finally, we performed data checking and initiated the data analysis.

### Statistical analysis

A two-part model was selected to analyze the determinants of formal care use and expenses. The two-part model is often used to model health cost data that include many zero observations because of a non-negligible proportion of non-users [25]. The probability of and expenses related to formal care use estimated in this model were defined as formal care use and expenses in 2015 (*t*). The explanatory variables were drawn from the 2014 SLTNAQ to examine the influence of a 1-year lag (*t-1*).

The first part of the model predicts the probability *I*_*t*_ of formal care use in year *t* (Formula 1) using a logistic model to determine the probability of observing a positive value. $X_{1(t-1)}^{i}$ refers to the status of the explanatory variables in the previous year (*t*-1), while *β*_1(*t*−1)_ is the estimation coefficient. The second part of the model seeks to explain formal care expenses, *M*_*t*_, conditional on nonzero formal care use in year *t* (Formula 2). $X_{2(t-1)}^{i}$ refers to the estimation of independent variables in the previous year (*t*-1), while *β*_2(*t*−1)_ represents the estimation coefficient and ε is the residual error.

$$I_{t}=X_{1(t-1)}^{i}\beta_{1(t-1)},\;\text{i}=1,\ldots ,\text{N};$$ (1)

$$M_{t}(y_{t}|I>0)=X_{2(t-1)}^{i}\beta_{2(t-1)}+\varepsilon ,\;\text{i}=1,\;\ldots ,\text{n};$$ (2)

Furthermore, due to the non-normal distribution of the raw formal care expenses, Box-Cox transformations of *y* indexed by *λ* were involved in the second part of the model, as shown in Formula 3.

$$y^{\left(\lambda \right)}=\{{}_{\log(y),if\lambda =0}^{(y^{\lambda }-1)/\lambda ,if\lambda \neq 0},y>0$$ (3)

When *λ* is equal to 0, a log transformation is applied. The parameter *λ* is determined by using profile log-likelihoods. In the second part of the model, 0.5 was contained in the 95% confidence interval of *λ*, $(\sqrt{spending}-1)/0.5$ was used as the response variable rather than the raw expenses.

All statistical analysis were performed using SAS 9.30. We set a significance level of 0.05 for hypothesis testing. For monthly income, median was taken as the cut off.

### Ethics statement

Ethics approval was obtained from the Shanghai Health Development Research Center’s ethics committee in 2013.

## Results

The proportion of participants who used formal care was 9.37% in 2015, and their average formal care expenses was 2417 yuan. The majority of the elderly adults (81.59%) chose informal care in 2014, among which 60.85% was provided by a spouse and 33.82% by children. Only 4.97% of respondents received formal care during that year (Table 1).

**Table 1:** Demographics, ADL scores, living arrangements, physical health status, and care type in 2014 (n = 7100).

Indicators in 2014	Values^a^
**Age**	72.12 ± 8.89
**Gender**	
**Male**	3319 (46.75)
**Female**	3781 (53.25)
**ADL scores**	19.17 ± 2.78
**Marital status**	
**With spouse**	5583 (80.04)
**Without spouse**	1392 (19.96)
**Children**	
**With children**	6637 (97.46)
**Without children**	173 (2.54)
**Living alone**	
**Yes**	726 (10.25)
**No**	6357 (89.75)
**Monthly income**	
**≥3500 yuan**	3832 (54.25)
**<3500 yuan**	3232 (45.75)
**Self-reported health status**	
**Healthy**	6464 (92.34)
**Unhealthy**	536 (7.66)
**Chronic diseases**	
**Hypertension**	3336 (46.99)
**Coronary heart disease**	965 (13.59)
**Stroke**	461 (6.49)
**Diabetes**	631 (8.89)
**Advanced tumor**	55 (0.77)
**Lower limb fracture**	55 (0.77)
**Dementia**	65 (0.92)
**Care type**	
**With formal care**	353 (4.97)
**With informal care**	5793 (81.59)
**Formal care fee**	1492.87 ± 1245.31

ADL, Activities of Daily Living.

^a^Values are means ± standard deviations (SDs) or numbers and percentages.

### Determinants of formal care use

The determinants of formal care use were tested in the first part of the model. The results for all indicators entered into the regression model were shown in Table 2. The odds of formal care use were found to increase with age. Females, those without a spouse, and those with a higher income had greater odds of formal care use in 2015. Notably, the odds of formal care use increased with ADL scores. With regard to chronic diseases, suffering from stroke, advanced tumor, lower limb fracture, and dementia led to much higher odds of formal care use. The odds ratios were highest for advanced tumor, followed by stroke. Individuals who received formal care in 2014 were inclined to continue using it in 2015; in contrast, participants who used informal care in 2014 were less likely to use formal care in 2015.

**Table 2:** Logistic regression analysis of odds ratios of formal care use.

Indicators	*OR*	*95% Wald CI*	*p-value*
**Age**	1.17	(1.15–1.19)	< .0001
**Gender (Female *vs*. Male)**	1.38	(1.12–1.70)	0.0022
**ADL scores**	1.03	(1.00–1.06)	0.0295
**Spouse(Without *vs*. with)**	1.87	(1.50–2.32)	< .0001
**Children (Without *vs*. with)**	1.74	(0.90–3.39)	0.1021
**Living alone (Yes *vs*. No)**	0.79	(0.59–1.08)	0.1368
**Income(≥3500 yuan *vs*. <3500 yuan)**	1.33	(1.09–1.63)	0.0055
**Self-reported health (Unhealthy *vs*. Healthy)**	1.18	(0.86–1.61)	0.3094
**Chronic diseases (Yes *vs*. No)**			
**Hypertension**	1.13	(0.92–1.39)	0.2354
**Coronary heart disease**	0.97	(0.76–1.23)	0.8112
**Stroke**	2.68	(2.04–3.53)	< .0001
**Diabetes**	1.23	(0.91–1.65)	0.1811
**Advanced tumor**	3.26	(1.46–7.28)	0.0039
**Lower limb fracture**	2.46	(1.15–5.27)	0.0203
**Dementia**	2.37	(1.23–4.56)	0.0096
**Care type in 2014 (Yes *vs*. No)**			
**Formal care**	2.29	(1.63–3.21)	< .0001
**Informal care**	0.64	(0.48–0.84)	0.0011

OR, odds ratio; CI, confidence interval; ADL, Activities of Daily Living.

To determine the association between income and formal care use, we analyzed the formal care utilization among the highest and lowest income groups that had the same ADL scores. We defined participants with an income of less than 2700 yuan (lower than *P*_*10*_) as the lowest income group, and those with an income of higher than 4500 yuan (upper than *P*_*90*_) as the highest income group. Among individuals with an ADL score below 20, the rate of formal care use in the lowest income group was 17.12%, whereas the rate reached 64.45% in the highest income group (*p*<0.05). Further analysis on the proportion of the income absorbed by formal care showed that, on average, 82.31% of the income in the lowest income group and 50.32% of the income in the highest group was absorbed by formal care services (*p*<0.05).

### Determinants of formal care expenses

The estimated coefficients (i.e., the variance explained by those variables) of the explanatory variables indicated that 24.30% of the variance in formal care expense could be explained by age, income, and formal care fee in 2014, as shown in Table 3. Specifically, formal care expense increased with age and income, but decreased with formal care fee in 2014.

**Table 3:** Regression results of the determinants of formal care expense.

Indicators	*b*	*S*_*b*_	*t*	*p-value*
**Intercept**	14.31	25.68	0.56	0.5780
**Age**	0.96	0.28	3.48	0.0006
**Gender (Female *vs*. Male)**	0.60	3.19	0.19	0.8502
**ADL scores**	-0.06	0.39	-0.15	0.8798
**Spouse (Without *vs*. with)**	2.43	3.36	0.72	0.4701
**Children (Without *vs*. with)**	-2.64	7.02	-0.38	0.7075
**Living alone (Yes *vs*. No)**	-3.88	4.33	-0.90	0.3705
**Income(≥3500 yuan *vs*. <3500 yuan)**	13.67	3.12	4.38	< .0001
**Self-reported health (Unhealthy *vs*. Healthy)**	3.17	3.74	0.85	0.3975
**Chronic diseases (Yes *vs*. No)**				
**Hypertension**	-0.68	3.27	-0.21	0.8353
**Coronary heart disease**	2.94	3.57	0.82	0.4106
**Stroke**	5.89	3.85	1.53	0.1272
**Diabetes**	-2.32	4.47	-0.52	0.6038
**Advanced tumor**	-12.83	14.08	-0.91	0.3631
**Lower limb fracture**	-11.04	9.21	-1.20	0.2319
**Dementia**	6.59	7.07	0.93	0.3521
**Formal care fee in 2014**	-0.01	0.00	-3.58	0.0004
**Informal care (Yes vs. No)**	5.85	3.33	1.76	0.0801

ADL, Activities of Daily Living.

## Discussion

The main focus of this study was to examine the determinants of formal care use and expenses using data from a large cohort of elderly adults in Shanghai. We found that greater use of formal care was associated with a higher ADL score, whereas ADL had no apparent influence on formal care expenses. This is surprising, given that ADL is widely regarded as an important predictor of formal care use [26, 27]. Unlike these past findings, we found that the older adults with higher ADL scores were more likely to employ formal care services. This suggested that the provision of formal care among elderly adults in Shanghai might not be needs-oriented. This is perhaps because the formal care system is still under development; the increase in odds associated with ADL might be due to a lack of proper assessment and professional guidance from society. Alternatively, elderly adults and their families tend to make decisions about formal care based on subjective judgments rather than real needs. Although the assessment procedure has been refined over the past several years, it still takes time for people, especially the elderly, to form habits of making decisions wisely.

The significant association between income and formal care also deserved our attention. The amount of formal care use and expenses were greater among those with a higher income. A number of studies have supported this positive relationship between economic status and formal care [10, 28], and it likely reflects an income-related inequity of unmet care needs. For example, two studies in China showed that the risk of having unmet needs was largely determined by elderly adults’ financial status [29, 30]. In the present study, the rate of formal care use in the highest income group was significantly greater than that in the lowest income group, even when they had the same ADL scores. This implied that the needs of certain individuals with low ADL scores were not completely satisfied because of these individuals’ limited economic means. Further, a higher proportion of income was absorbed by formal care services in the lowest income group, meaning that low-income families, compared to high-income families, bear an even heavier financial burden in caring for elderly adults. As an indicator of socioeconomic status, income is closely related to other sociodemographic factors such as occupation and marital status, which also have been found to influence health and unmet care needs [31].

Interestingly, care type in 2014 had a substantial effect on formal care utilization in 2015. Formal care use in 2014 predicted its use in 2015, suggesting that it was difficult to live without formal care once elderly adults had begun using it. This finding illustrated that elderly adults who used formal care in 2014 had some form of dependency that made it difficult to return to independent living [32]. Given this, the decision to provide and use formal care should be deliberated at both the government and individual levels.

In line with previous studies, older age predicted greater use and expenses of formal care, which was similar to the findings in western populations [33]. The odds of formal care use increased 0.17 times per year of age. Thus, it was estimated that the odds would increase by 1.19 times for every 5-year increase in age, and 3.81 times for every 10-year increase. We similarly found that women were more likely to use formal care, which was consistent with previous research [34]. Women tend to have greater longevity and are traditionally seen as the main caregivers [35]. This was similarly true that in Shanghai, where the life expectancy for women was approximately 5 years longer than that for men in 2015. In this case, when women (i.e., the primary caregivers) themselves need support, their families may be more likely to hire formal caregivers. Additionally, individuals without a spouse have been found to have a greater likelihood of formal care utilization. This is perhaps because the spouse who makes up the primary source of informal care in each family. Furthermore, previous studies have confirmed that elderly adults turned to paid help only when informal care is not available [36]. The strong positive relationships between certain chronic diseases and formal care use suggested that more attention should be directed to the prevention of such diseases.

There are some limitations of this study. First, the formal care fee variable in our data was self-reported, which may mean that the results for this variable do not represent the true picture. To obtain more realistic estimations, we suggested that an average market price for a set amount of time should be added to the future waves of the SLTNAQ. Second, we recommended to carry out a stratified analysis by gender in future studies, since our regression results suggested that females are more likely to consume formal care services. Third, all respondents in this study were recruited from one sub-district in central Shanghai. Generalizing our findings to the overall situation in Shanghai probably requires caution.

## Conclusion

This study found that formal care use in Shanghai did not correlate with ADL, but did relate to income. Furthermore, suffering from certain kinds of chronic diseases led to increased odds of formal care use. Therefore, the need to revise the unified assessment of long-term care needs appeared to be extremely urgent. By developing this instrument, the government could offer gradient formal care services linked with ADL. Our findings suggested that more attention should be paid to the older adults of lower economic status. Financial support and public formal care could be given to low-income elderly to ensure that they have adequate access to formal care services. Establishing a long-term care insurance system may be a valid approach to cope with the increasing demand for formal care and alleviate burden on the family in the long run [37, 38].

## Supporting information

S1 Appendix Raw data. (SAV)

S2 Appendix Database instruction. (DOC)
