# Supplementary material for: Determinants of formal care use and expenses among in-home elderly in Jing’an district, Shanghai, China
Source: PLoS One. 2017 Apr 27;12(4):e0176548. doi: 10.1371/journal.pone.0176548 (PMC5407841; doi:10.1371/journal.pone.0176548)
Supplement: S2 Appendix — (DOC) [file pone.0176548.s002.doc]

**Database Instruction**

This study was embedded within the Shanghai Long-Term Care Needs Assessment Questionnaire (SLTNAQ), a cohort survey conducted in Jing’an district, Shanghai. Jing’an had the highest degree of aging population among all districts in Shanghai, that is, 33.9% of the total household population aged above 60 in 2015.The SLTNAQ is a longitudinal survey, designed to examine the long-term care needs of the elderly. The variables assessed by the SLTNAQ include demographic indicators (i.e. age, gender, and income), living arrangements, ADL scores, IADL scores, mental health status, cognitive status, physical status and a clinical diagnosis (provided by general practitioners). From among five sub-districts in Jing’an, Jiangning Road was randomly selected and sampled. Among the 17000 elderly living in Jiangning Road, 8500 residents aged over 60 were randomly selected and observed in 2014. Of these, 8428 in-home individuals responded to the questionnaire. 7100 of them were followed up in 2015.

The database uploaded is raw data, consisting of 7100 participants observed in 2014 and followed up in 2015. 21 variables are included in the dataset. Therefore, in total, 7100*21 data is uploaded. Among the 21 variables, *Age_2014, Gender_2014, ADL_2014, Formalcare_withORwithout_2014, Informalcare_withORwithout_2014, Spouse_withORwithout_2014, Children_withORwithout_2014, Selfreportedhealth_2014, Livealone_yesORno_2014, Monthlyincome_2014, Hypertension_yesORno_2014, Coronary_heart_disease_yesORno_2014, Stroke_yesORno_2014, Diabetes_yesORno_2014, Advanced_tumor_yesORno_2014, Lower_limb_fracture_yesORno_2014, Dementia_yesORno_2014, Formalcarefee_2014* are derived from the survey data in 2014, while *Informalcare_withORwithout_2015, Formalcare_withORwithout_2015, Formalcarefee_2015* are derived from the survey data in 2015.

For further data information, please contact Hansheng Ding ([dinghansheng@hotmail.com](mailto:dinghansheng@hotmail.com), [dinghansheng@smhb.gov.cn](mailto:dinghansheng@smhb.gov.cn)).
